# Tracking clonal dynamics of CD8 T cells and immune dysregulation in progression of systemic lupus erythematosus with nephritis

**DOI:** 10.1038/s12276-025-01504-2

**Published:** 2025-08-01

**Authors:** Seung-Jun Paek, Hye-Soon Lee, Ye Ji Lee, So-Young Bang, Dongju Kim, Bo-Kyeong Kang, Dae Jin Park, Young Bin Joo, Mimi Kim, Hyunsung Kim, Sung Yul Park, Woong-Yang Park, Tatsuki Abe, Takahiro Itamiya, Yasuo Nagafuchi, Kazuyoshi Ishigaki, Keishi Fujio, Kyu-Tae Kim, Sang-Cheol Bae

**Affiliations:** 1 Department of Physiology, School of Medicine, Ajou University, Suwon, Republic of Korea; 2 Department of Biomedical Sciences, Graduate School of Ajou University, Suwon, Republic of Korea; 3 Department of Rheumatology, Hanyang University Hospital for Rheumatic Diseases, Seoul, Republic of Korea; 4 Hanyang University Institute for Rheumatology Research, Seoul, Republic of Korea; 5 Hanyang Institute of Bioscience and Biotechnology, Seoul, Republic of Korea; 6 GENINUS Inc., Seoul, Republic of Korea; 7 Department of Translational Medical Science, Graduate School of Biomedical Science and Engineering, Hanyang University, Seoul, Republic of Korea; 8 Department of Radiology, College of Medicine, Hanyang University, Seoul, Republic of Korea; 9 Department of Pathology, College of Medicine, Hanyang University, Seoul, Republic of Korea; 10 Department of Urology, College of Medicine, Hanyang University, Seoul, Republic of Korea; 11 Samsung Genome Institute, Samsung Medical Center, School of Medicine, Sungkyunkwan University, Seoul, Republic of Korea; 12 Department of Allergy and Rheumatology, Graduate School of Medicine, The University of Tokyo, Tokyo, Japan

**Keywords:** Autoimmunity, Lupus nephritis

## Abstract

The fluctuating nature of disease activity in systemic lupus erythematosus (SLE), alternating between flares and remissions, poses substantial challenges for its effective management. The use of current biomarkers for monitoring SLE is limited in clinical settings owing to insufficient comprehension of the complex immune involvement underlying the disease course. Here, therefore, we profiled peripheral blood mononuclear cells at both stable and exacerbation states (total of *n* = 19) from six patients with SLE and 32 healthy donors using integrated single-cell RNA and T cell receptor (TCR) sequencing. To validate our findings, we analyzed two independent external datasets: bulk RNA sequencing and TCR data from 79 controls and 62 patients with SLE and single-cell RNA sequencing data from 99 healthy controls and 162 patients with SLE. Our analysis revealed cell type-specific activation of interferon-related genes in SLE grouped into four clusters, with elevated activity in disease-associated immune cells. Among these, atypical B cells associated with autoantibody production exhibited distinct differentiation patterns compared with conventional memory B cells, driven by heightened interferon signaling in SLE. Notably, clonal expansion of effector CD8 T cells emerged as a key driver of disease exacerbation, as indicated by reduced TCR diversity. Specific CD8 T cell clonotypes expanded during flare states, transitioning to effector phenotypes that exhibited heightened cytotoxicity and amplified interferon signaling, strongly correlating with tissue damage and flare severity. Our findings establish a critical link between interferon-driven mechanisms and cytotoxic T cell dysfunction in SLE flares, offering potential targets for therapeutic intervention and predictive biomarkers.

## Atypical B cells and CD8 T cells fuel lupus exacerbations

Systemic lupus erythematosus (SLE) is a complex autoimmune disease that affects various organs and presents differently in each patient. This study addresses the challenge of understanding the variability of SLE by examining immune cells at a detailed level. The authors used single-cell RNA sequencing to analyze blood samples from six patients with SLE and healthy controls. The study focused on peripheral blood mononuclear cells, which are crucial for the immune response. By examining these cells individually, the researchers identified over 20 distinct cell types involved in SLE. They found that certain immune cells, such as CD8 T cells, were active during disease flare-ups, which are periods of increased symptoms. The results showed that specific gene patterns in these cells could predict disease activity and help classify patients into subtypes. This understanding could lead to more personalized treatments for SLE.

This summary was initially drafted using artificial intelligence, then revised and fact-checked by the author.

## Introduction

Systemic lupus erythematosus (SLE) is a paradigmatic example of a systemic autoimmune disease involving a diverse array of immune cells in its pathogenesis. Its clinical manifestations range from mild skin rash and arthritis to severe, life-threatening organ complications. The variable nature of SLE, with sporadic flares and remissions, reflects deep heterogeneity at the cellular and molecular levels, influenced by patient-specific genetic and molecular differences^1^. The primary goals in SLE management are to reduce disease activity, prevent organ damage and improve quality of life^2^. However, clinical heterogeneity in pathophysiological presentations complicates treatment as responses can vary substantially among patients. Given the variability of SLE, a personalized approach based on patient stratification is essential for effective disease management^3^.

Profiling peripheral blood mononuclear cells (PBMCs) provides a potent, noninvasive tool for understanding the altered signaling pathways of the multiple immune cell types involved in the pathogenesis of disease. The analysis of gene expression profiles in bulk immune cells from patients with SLE reveals distinct phenotypic traits of their subsets^4,5^, potentially enhancing patient stratification and consequently improving clinical outcomes with better treatment options^6,7^. However, approaches that analyze heterogeneous immune cells en masse as a pooled population may not fully capture the complete immune landscape of SLE, potentially overlooking critical populations that are essential for understanding the disease pathophysiology.

Here, to address this limitation, single-cell RNA sequencing (scRNA-seq) was employed to profile individual PBMCs in patients with SLE^8–11^. The analysis of immune cell heterogeneity within and among patients with SLE revealed several key findings: (1) the identification of >20 distinct cell subsets actively involved in disease pathogenesis, (2) global and cell type-specific expression of interferon gene signatures across various immune cells and (3) differences in certain cellular compositions in comparison to controls, such as a decrease in naive CD4 T cells and an increase in repertoire-restricted CD8 T cells. Notably, Perez et al. proposed that cell type-specific expression patterns precisely predict case–control status and enable the stratification of patients into molecular subtypes^11^. Furthermore, multiple studies have suggested the potential roles of specific cell components in SLE pathogenesis, such as double negative switched memory B cell (CD27^−^IgD^−^CXCR5^−^CD11c^+^DN2 cells) and atypical B cell (ABC) populations (CD11c^+^TBX21^+^)^8,10–12^. These studies also demonstrated that a subset of CD8 T cells, such as CD8 effector/memory T cells with elevated expression levels of cytotoxic genes, undergo clonal expansion and may have a substantial influence on disease progression.

Despite these valuable insights from scRNA-seq studies, the fluctuation of immune cell states during flare-ups and remission remains underexplored. Given the variability in immune responses among patients with SLE, long-term immune monitoring is essential for understanding pathophysiological dynamics, the early detection of flares and personalized refinement of treatment regimens. Accordingly, this study aims not only to investigate the contrasting cellular and molecular profiles of patients with SLE against those of controls, but also to track their changes throughout the disease course by analyzing longitudinal PBMC scRNA-seq data. Furthermore, we integrate immune repertoire profiling with gene expression profiles to explore the crucial role of autoantigen immune responses in SLE pathogenesis. This combination could enhance our understanding of antigen-specific immune cell dynamics, offering insights into the underlying mechanisms of SLE flare-ups.

## Materials and methods

### Study design

This study enrolled six patients who were diagnosed with SLE at Hanyang University Hospital for Rheumatic Diseases. These patients were ≥18 years old and met either the 2012 Systemic Lupus International Collaborating Clinics classification criteria or the 2019 European League Against Rheumatism/American College of Rheumatology classification criteria^13,14^. Each patient experienced at least one flare state with an increased Systemic Lupus Erythematosus Disease Activity (SLEDAI) score during the disease course^15^. A flare was defined as an increase in Physician Global Assessment by ≥1.0 and treatment intensification. Two of the six patients experienced two states of flares followed by remission. All individuals initially presented with lupus nephritis in conjunction with a lupus flare-up. Clinical data were collected both before and immediately after the flare. Before experiencing a flare, all patients were prescribed an oral immunosuppressant, such as mycophenolate mofetil, or a calcineurin inhibitor, either alone or combined with low-dose corticosteroids. Additionally, we included 32 Korean participants as controls from the Asian Immune Diversity Atlas, which is a part of the Human Cell Atlas–Asia^16^.

### Sample preparation and library construction

Blood samples were collected from our cohort and PBMCs were isolated and cryopreserved. Frozen cells were then resuspended in a 1× PBS solution and filtered through a 40 µm filter. A small portion of the cell solution was mixed with acridine orange and propidium iodide (AO/PI) and placed on a cell-counting slide to assess viability using the LUNA-FL automated fluorescence cell counter. Cells with a viability greater than 70% were used for sequencing.

We utilized the Chromium Next GEM Single Cell 5′ kit v2 and the Chromium Next GEM Chip K Single Cell kit to generate a barcoded 5′ single-cell library (10X Genomics Chromium). Each cDNA library that passed quality control (on an Agilent Bioanalyzer) was sequenced on an Illumina NovaSeq6000 or NextSeq500. Immune repertoire sequencing was performed using cDNA synthesized through the nested-PCR enrichment method. T cell receptor (TCR) libraries were generated using the Chromium Single Cell Human TCR Amplification kit (10X Genomics). Before sequencing, each library underwent a quality control assessment on an Agilent Bioanalyzer.

### Flow cytometry and cell sorting

PBMCs were incubated with fluorochrome-conjugated antibodies at 4 °C for 20 min to label surface markers. Viability staining was performed using the Live/Dead Cell Stain kit (Invitrogen, L34972) to exclude dead cells. Flow cytometry was carried out on an LSR II instrument (BD Biosciences), and the analysis was conducted with FlowJo software v10.10 (Treestar). Antibody clones and their experimental suitability were validated through prepurchase review of flow cytometry plots and peer-reviewed literature citations. Serial dilution titrations of each antibody were performed on PBMCs to determine the optimal staining concentrations and validate that observed cell population proportions aligned with published references.

The following directly conjugated antibodies were used to identify cell markers of human CD8 T cells (clone, manufacturer): mouse anti-human CD4-BV510 (SK3, BD Biosciences), mouse anti-human CD3-BV605 (UCHT1, BD Biosciences), mouse anti-human CD8-FITC (RPA-T8, BD Biosciences), mouse anti-human CCR7-PerCP-Cy5.5 (150503, BD Biosciences), mouse anti-human CD14-PE-TR (61D3, eBioscience), mouse anti-human CD19-PE-TR (HIB19, eBioscience) and mouse anti-human CD45RA-APC/Cyanine7 (HI100, Biolegend).

### External data sources included in the study

To consolidate our findings, we adopted two independent external datasets. First, bulk RNA and TCR sequencing data from 28 immune cell types, including those from 79 healthy controls and 62 patients with SLE, were obtained from the National Bioscience Database Center (NBDC) Human Database (accession number E-GEAD-397)^17^. Matched TCR sequencing data and disease activity information for bulk RNA-seq data were provided with the approval of the Ethical Committee of The University of Tokyo. When immune cell subsets belonged to broader categories, expression levels were averaged across these subsets. Second, PBMC scRNA-seq data from 99 healthy controls and 162 patients with SLE (GSE174188)^11^ were included in the analysis. These data were integrated, processed using a pipeline consistent with that applied to our study samples. 10 immune cell types were annotated based on markers identified in the original study and those determined through our analyses, ensuring consistency for cross-study comparisons.

### scRNA-seq data quality control and preprocessing

scRNA-seq data were processed using Cell Ranger (v6.0.0)^18^ to align against the GRCh38 human reference genome provided by 10X Genomics. This step involved identifying and counting unique molecular identifiers (UMIs) and filtering cells to ensure data integrity. The reference genome utilized was obtained from 10X Genomics (Human reference GRCh38 (ref. ^19^)).

We utilized Seurat (v4.1.1)^20^ in R (v4.1.1.) to select cells with more than 500 genes and <5% mitochondrial gene content and platelet with marker gene expression (*PPBP*, *PF4* and *NRGN*). Additionally, Rscrublet (v0.1.0, R port of scrublet^21^) was utilized to remove the doublet, and any potential doublet was manually removed based on marker expression. After cell filtering and expression normalization, a total of 155,923 cells remained in the final dataset.

During the variable feature extraction step for dimensional reduction, we excluded blacklisted genes to prevent undesired influence. These included the following: (1) variable genes of α/β TCR sequences, (2) variable genes of immunoglobulin sequences, (3) ribosomal protein genes, (4) mitochondrial genes and (5) dissociation stress signature genes^22^. After performing principal component analysis, the potential batch effect caused by sample difference was corrected using harmony (v0.1.0) to eliminate technical discrepancies between samples^23^. Subsequently, Uniform Manifold Approximation and Projection (UMAP) and clustering were performed on the harmony space to facilitate embedding and clustering, thereby establishing a solid foundation for robust downstream analyses.

### Cell type identification

The marker genes for clusters were identified by comparing differently expressed genes between clusters and the most abundantly expressed genes for each cluster with known markers for each cell type. These cell types were validated using reference studies^24–26^. Subsequently, each subtype within the main cell type was distinguished by extracting and reclustering the global cell types. Cells annotated as ‘cycling’ were then divided among each lymphoid cell subtype (CD4 T, CD8 T and natural killer (NK)) through higher-resolution clustering based on the same marker genes associated with each lymphoid cell subtypes. Additionally, distinct γ/δ TCR sequence gene expression was used for γδ T cells (gdT). Details of the marker gene list are described in Supplementary Table 2.

To validate the identified cell types at both global and subtype levels, an automated annotation from the reference-based mapping tool Azimuth (v0.4.6) was employed^20^. A PBMC dataset containing 161,764 cells, produced from the same study^20^, was applied as a reference. Cells were assigned to a specific cell type that exhibited the highest probability based on gene expression profiles from the reference. The agreement rate for the assigned cell type was quantified by the proportion of our annotated cells that corresponded to similar cell types in the reference dataset.

### Differential expression analysis

Differential expression analysis among cell types in scRNA-seq data was performed using the FindMarkers function in Seurat, utilizing the model-based analysis of single-cell transcriptomics method. The genes with fold change >1.25 and adjusted *P* value < 10^−6^ by Benjamini–Hochberg correction were considered as being significantly differentially expressed.

The gene sets were generated from curated collections in MSigDB^27^ and Gene Ontology. Gene set scores in scRNA-seq data were calculated by determining the average expression levels of aggregated gene sets using the AddModuleScore function in the Seurat package, with scores normalized using *z*-score transformation. For bulk RNA-seq data, gene set scores were computed using Gene set variation analysis (GSVA, v1.42.0)^28^, followed by *z*-score normalization. Further details regarding the gene set can be found in Supplementary Table 3.

### Cell differentiation trajectory inference

Cellular trajectories of differentiation for B cells and CD8 T cells were inferred using monocle3 (v1.0.0)^29^ on UMAP embedding. In this analysis, we excluded plasma cells from B cells and cycling CD8 T cells owing to the distinct gene expression signature compared with other subtypes. The root node was specified within clusters of naive subtypes (naive B and CD8 T cells) based on the concept of immune cell transition. Pseudotime was calculated based on this cell ordering.

### Regulon network analysis

To examine the regulon network via transcription factor (TF) activity, we utilized SCENIC (v1.3.1)^30^ and pySCENIC. This approach analyzes the co-expression of TFs and their putative target genes. We started with a raw count matrix to construct the co-expression network, denoising on genes expressed in more than 1% of the total cells. For the enrichment analysis of gene signatures, we employed motifs from the cisTarget Human motif database v10 (ref. ^31^). The regulon activity for each cell was determined by calculating the average normalized expression of the putative target genes.

### Cell–cell communication analysis

Intercellular communication was explored using CellChat (v1.6.1)^32^. This approach enables the inference of cellular interactions by mapping potential ligand–receptor pairs across different cell types. Interactions were determined based on the expression of ligand genes in one cell type and receptor genes in other cell types, with the strength of interaction weighted by communication probability. This analysis provides insights into the intricate signaling networks within the cellular environment.

### Single-cell TCR sequencing data processing and clonotype analysis

TCR sequencing data were processed with Cell Ranger (v6.0.0)^18^ for mapping TCR reads against the V(D)J reference genome, counting UMIs and cell filtering, as provided by 10X Genomics. A reference genome was obtained from the cell ranger pipeline (Human V(D)J reference GRCh38 (ref. ^33^)). To assign TCR chain CDR3 sequences to the corresponding cells within the scRNA-seq dataset, we usedscRepertoire (v1.5.2)^34^. During this step, the following filtering criteria were applied to minimize noise: (1) only high-confidence sequences were retained, (2) if a cell had more than one chain of the same type, only the chain with the highest UMI count was retained, (3) only cells with paired alpha and beta chain sequences were retained and (4) cells with the same V,D,J genes in both chains were assigned to the same clonotype.

To evaluate T cell expansion, we measured TCR clonotype diversity using Shannon’s entropy index. The similarity between TCR clonotypes in each group was measured using the Morisita–Horn similarity index. Significantly expanded clonotypes during flare states were identified by comparing clonotype sizes (that is, the number of cells per clonotype) between pre-flare and flare states. Clonotypes exhibiting at least a twofold increase in size during flare or clonotypes absent at baseline but present during flare state were classified as expanded. To minimize spurious signals, only clonotypes showing proportional expansion and represented by ≥3 cells during the flare state were included in the analysis.

### Statistical analysis

All values are presented as the means ± s.d. No statistical method was used to predetermine the sample size. Adjusted *P* values were reported to correct for multiple testing via the Benjamini‒Hochberg procedure, ensuring control over false discovery rates. Statistical significance was defined as a *P* value <0.05 for all tests. All the statistical analyses were performed using R statistical software.

## Results

### Profiling the immune cell landscape in SLE

**Fig. 1:**
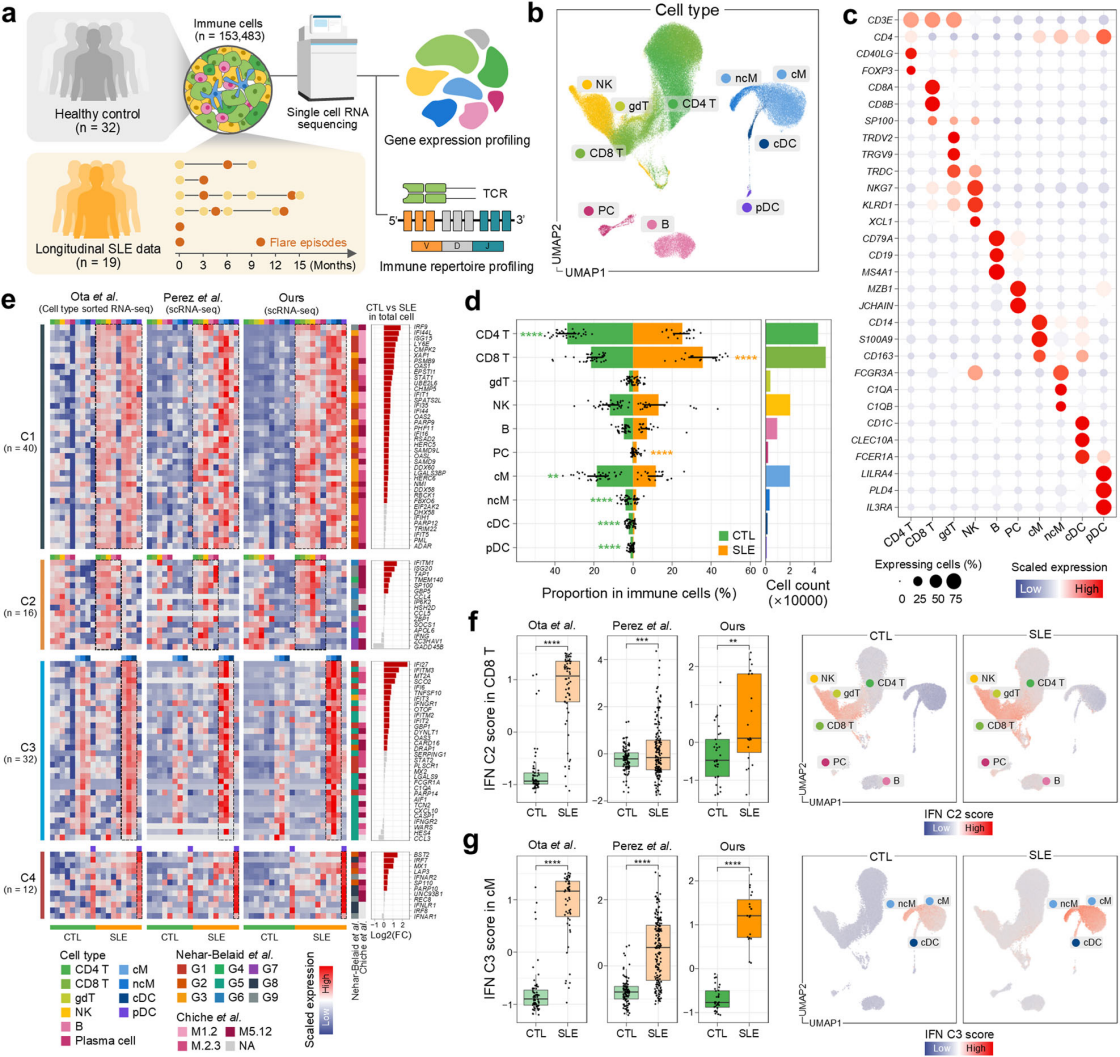
Characterization of the immune landscape in SLE. **a** A schematic of the single-cell transcriptome with TCR sequence profiling of immune cells in PBMCs from healthy controls (CTL) and patients with SLE. **b** UMAP visualization of immune cell types. **c** Scaled average expression of marker genes across ten immune cell types, with the dot size indicating the proportion of expressing cells and the color gradient indicating expression levels. **d** A bar plot showing the cell type proportions by disease state (left) and the total number of immune cells (right). The lines on the bar indicate the interquartile range (IQR), spanning from the 25th percentile (Q1) to the 75th percentile (Q3). Statistically significant increases are marked with orange stars for SLE and green for CTL. **e** A heat map of 100 IFN-related genes across different cell types across three different studies. log_2_ fold changes are shown, with genes significantly overexpressed in SLE marked in red (right). **f**,**g** A comparison of the IFN C2 score in CD8 T cells (**f**) and the IFN C3 score in cM (**g**) across controls and patients with SLE. The box plots show the distribution of IFN scores from three different studies (left). The box represents the IQR, spanning from Q1 to Q3, with the line inside the box indicating the median. The whiskers extend to the smallest and largest values within 1.5 times the IQR. UMAP visualization of IFN scores across immune cells (right). The color gradient indicates low to high scores. **P < 0.01, ***P < 0.001 and ****P < 0.0001.

To dissect the multicellular ecosystem of SLE and understand the development of disease progression, we employed scRNA-seq on PBMCs from 32 healthy controls and 19 samples from six patients with SLE (at different time points throughout the disease course), four of whom have experienced at least one cycle of flare and remission (Fig. 1a). SLEDAI scores ranged from 4 to 14 before flare-up and increased from 9 to 19 during flare states (Supplementary Fig. 1 and Supplementary Table 1). All patients demonstrated renal involvement, evidenced by increased proteinuria or decreased estimated glomerular filtration rate (eGFR), along with the presence of anti-double-stranded DNA (dsDNA). Furthermore, we observed significant correlations between the SLEDAI score and anti-dsDNA titer, as well as proteinuria (Supplementary Fig. 1a). Additionally, clinical data indicated elevated proteinuria levels during flare states compared with pre-flare states (Supplementary Fig. 1b).

Overall, we analyzed 153,483 cells after filtering out putative doublets and poor-quality cells. Integrating gene expression profiles across samples and unsupervised clustering of cells with canonical marker inspection enabled us to identify 11 major cell types. These cell types were visualized in a UMAP space: CD4^+^ T cells (CD4 T), CD8^+^ T cells (CD8 T), NK cells (NK), gdT, B cells (B), plasma cells (PC), classical monocytes (cM), nonclassical monocytes (ncM), conventional dendritic cells (cDC) and plasmacytoid dendritic cells (pDC) (Fig. 1b,c and Supplementary Table 2). Our assigned cell types demonstrated a significant level of concordance with the automated annotation result from the reference-based mapping tool Azimuth^20^ (88.78 ± 21.36% and 68.34 ± 27.40%, mean ± s.d. of agreement rates for the cell types with the matched label at a global and subtype level, respectively) (Supplementary Fig. 2).

We observed a significant difference in the composition of cell types between SLE and controls. The SLE samples showed a marked decrease in CD4 T cells, monocytes, cDCs and pDCs, alongside a pronounced increase in CD8 T and B cells (Fig. 1d and Supplementary Fig. 3), which was confirmed by external scRNA-seq data (Supplementary Fig. 4)^11^. In addition, consistent with previous findings^35,36^, a significant decrease in the CD4 to CD8 T cell ratio in the SLE samples was observed (Supplementary Fig. 3d).

### Comprehensive analysis of IFN-stimulated genes in SLE

To understand the molecular signatures enriched in SLE in comparison with controls, we performed differential expression analysis on aggregated cells overall. Expectedly, we detected type I IFN signaling-related genes (such as *IFITM1*, *ISG15* and *IFI27*) with higher expression levels in SLE (Supplementary Fig. 5a). We yielded concordant results on gene set enrichment analysis, showing enriched IFN-related signaling pathways in SLE (Supplementary Fig. 5b), consistent with previous studies demonstrating elevated activity of IFN signaling pathways in SLE pathophysiology^37,38^.

To explicitly probe the IFN module in a cell type-specific manner, we further investigated the previously reported 100 IFN-related genes (Supplementary Tables 3 and 4)^10^. We classified them using *K*-means clustering and determined *K* = 4 was the optimal number of clusters based on the silhouette score (Supplementary Fig. 6a,b) and its shared expression pattern within specific cell types (Supplementary Fig. 6c), not only in our scRNA-seq data but also in two independent external datasets of cell type-sorted bulk RNA-seq^17^ and scRNA-seq^11^ data, respectively (Fig. 1e, Supplementary Fig. 6d and Supplementary Table 5). Cluster C1 exhibited upregulation across most immune cell types, including a broad range of IFN-stimulated genes (*IFI44*, *IFI16*, *IFI35* and *ISG15*) and IFN-related signaling pathways such as the JAK/STAT pathway (*STAT1* and *IRF9*)^39^. Cluster C2 was related to antigen-receptor signaling in lymphocytes (*IFITM1*, *SP100*, *ISG20* and *TAP1*)^40,41^ and chemokine regulation involved in the recruitment and activation of lymphocytes (*CCL4*, *CCL5* and *SOCS1*)^42^, and showed enhanced expression predominantly in lymphocytes. Particularly, we observed significantly higher cluster C2 module scores in CD8 T cells of SLE in all three independent datasets (Fig. 1f). Next, cluster C3 included genes associated with IFN stimulation (*IFI6*, *OAS3* and the IFIT family) and cytokine production by myeloid cells (*CXCL10*, *SERPING1*, *TNFSF10* and *CASP1*)^43^ with upregulated expression in monocytes and cDCs. Significant augmented cluster C3 module scores in cM cells of SLE were identified in all three independent datasets (Fig. 1g). Notably, cluster C4 was upregulated in pDCs with genes that are associated with type I IFN production (*IRF7*, *IRF8* and *BST2*)^44,45^. These classifications demonstrated high functional concordance with two previous modular approaches^10,46^ (Fig. 1e and Supplementary Fig. 6e).

### Identification of B lymphocyte subtypes with enhanced type I IFN responses

**Fig. 2:**
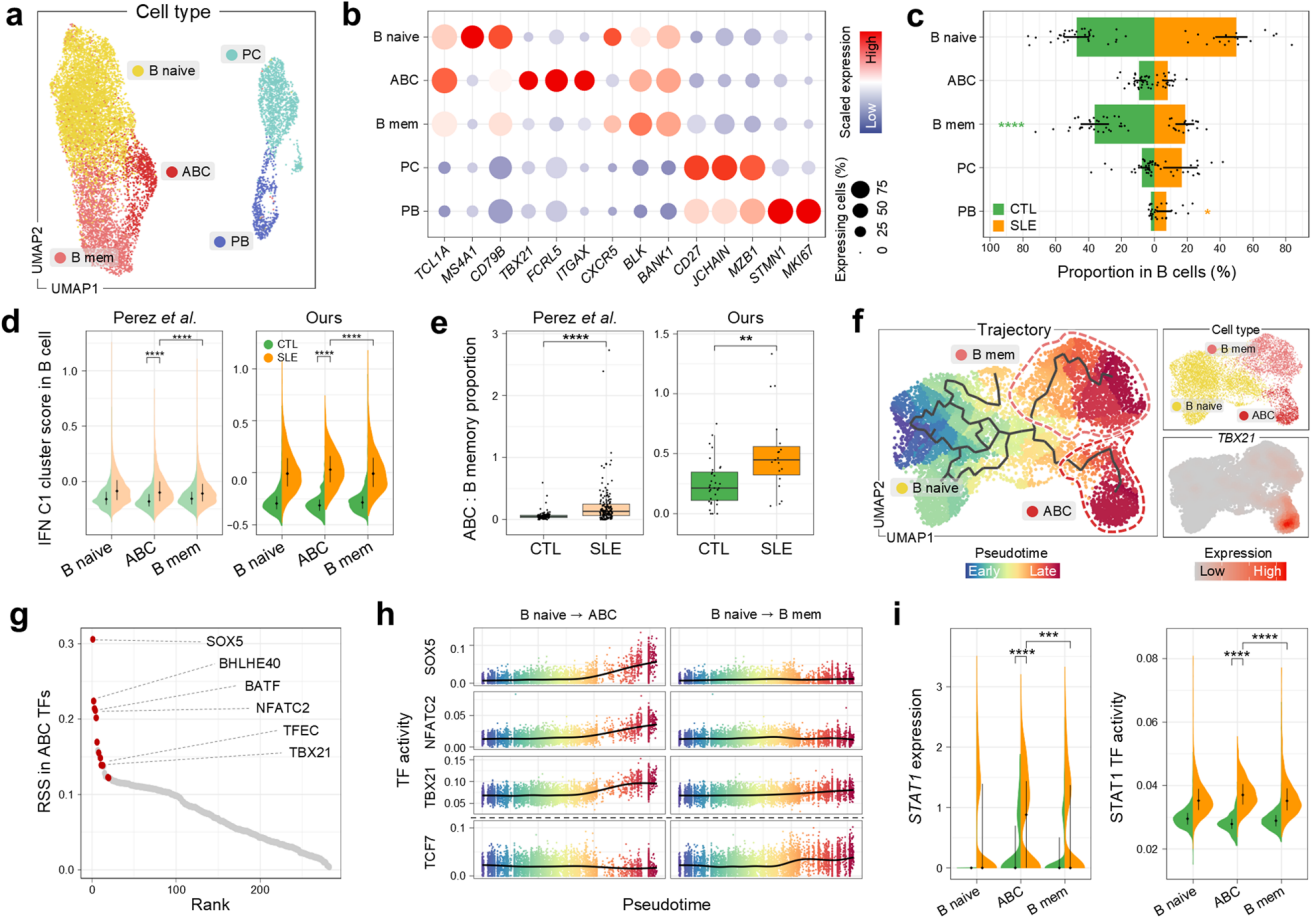
Novel subtype of atypical B cell with elevated IFN pathway. **a** A UMAP visualization of B cell subtypes: ABC, memory B cells (B mem), naive B cells (B naive), plasma cells (PC) and plasmablasts (PB). **b** Scaled average expression of marker genes across B cell subtypes, with the dot size indicating the proportion of expressing cells and the color gradient indicating the expression levels. **c** The proportion of B cell subtypes by disease state. The lines on the bar indicate the IQR, spanning from Q1 to Q3. Statistically significant increases are marked with orange stars for SLE and green for CTL. **d** Violin plots showing the distribution of IFN C1 cluster scores in B cell subtypes for controls (CTL) and patients with SLE in two studies: Perez et al.^11^ (left) and our study (right). The lines within the violin indicate the IQR, spanning from Q1 to Q3. **e** The ratio of ABC to B mem within total B cells, categorized by disease. The box represents the IQR, spanning from Q1 to Q3, with the line inside the box indicating the median. The whiskers extend to the smallest and largest values within 1.5 times the IQR. **f** A UMAP plot illustrating the inferred differentiation trajectory of non-plasma B cells, with color-coding from early (blue) to late (red) stages (left). UMAP plots for subtypes and *TBX21* expression levels (right). The color gradient indicates the expression level. **g** The regulon specificity score (RSS) of TFs in ABC. TFs are sorted by RSS rank and ABC-specific TFs, appearing exclusively in the top 30 for ABCs, are highlighted in red. **h** TF activity changes across pseudotime for B cell subtypes in B naive to ABC (left) and B naive to B mem (right). The color gradient represents the progression through pseudotime, with the lines indicating the trend of TF activity. **i** Violin plots of STAT1 expression and TF activity in non-plasma B cell subtypes. Lines within the violin indicate the IQR, spanning from Q1 to Q3. ***P < 0.001 and ****P < 0.0001.

To better understand B cell-mediated immune responses and their skewed functions in SLE, we first reclustered B cells into five subpopulations (Fig. 2a) based on established marker genes (Fig. 2b). Subcellular compositional analysis showed a substantial increase of plasma cells and plasmablasts, both of which function as antibody-secreting cells, in SLE (Fig. 2c), consistent with earlier findings^10,47^.

Of note, we identified ABCs that expressed *TBX21* (T-bet) and *ITGAX* (CD11c) with reduced *CXCR5* expression (Fig. 2b). While memory B cells typically represent a more controlled immune response, ABCs are often associated with increased autoantibody production and disease activity in autoimmune conditions^48,49^. To determine the dysregulated status of B cell-mediated immune responses, we assessed IFN module activity using IFN C1 cluster genes, which were globally elevated across all cell types in association with IFN-related signaling pathways such as the JAK/STAT pathway (Fig. 1e). We observed that ABCs exhibited the most pronounced IFN module scores among B cell subtypes (Fig. 2d), suggesting that the aberrant behavior of ABCs may be driven by the heightened IFN stimulation characteristic of SLE.

We next asked how the functional balance between autoreactive and regulated immune responses was distorted in SLE. The ratio of ABCs to memory B cells was prominently elevated in SLE across both scRNA-seq datasets (Fig. 2e and Supplementary Fig. 7), implying that SLE may enhance the transition of B cells toward antibody-secreting cells through unconventional differentiation pathways. To further explore how B cells are differentiated into these two functionally distinct subsets, we reconstructed the differentiation trajectory by inferring cell orders based on similarities of their expression patterns^29^. Although ABCs and memory B cells both originated from naive B cells, their differentiation paths bifurcated with distinct gene expression patterns (Fig. 2b, f). The downregulation of *CXCR5*, a key receptor for follicle homing, in ABCs supported the notion that ABCs mature in extrafollicular regions, whereas conventional B cells mature within the germinal centers of follicular regions^48^. These two distinct fates were further highlighted by TF analysis. ABCs exhibited activation of TFs such as TBX21, SOX5, NFATC2 and BATF (Fig. 2g), and notably their activities increased with differentiation (Fig. 2h). In contrast, other TFs such as TCF7, which regulate memory B cell differentiation, were not activated during ABC differentiation (Fig. 2h), indicating distinct regulatory pathways for these subtypes. Additionally, the gene expression and TF activity of STAT1 elevated by IFN stimulation are significantly higher in ABCs from patients with SLE (Fig. 2i). These changes in TFs, including STAT1 activity, along the ABC differentiation trajectory were supported by an external SLE scRNA-seq dataset (Supplementary Fig. 7).

### Altered behavior of T lymphocytes in SLE pathogenesis

**Fig. 3:**
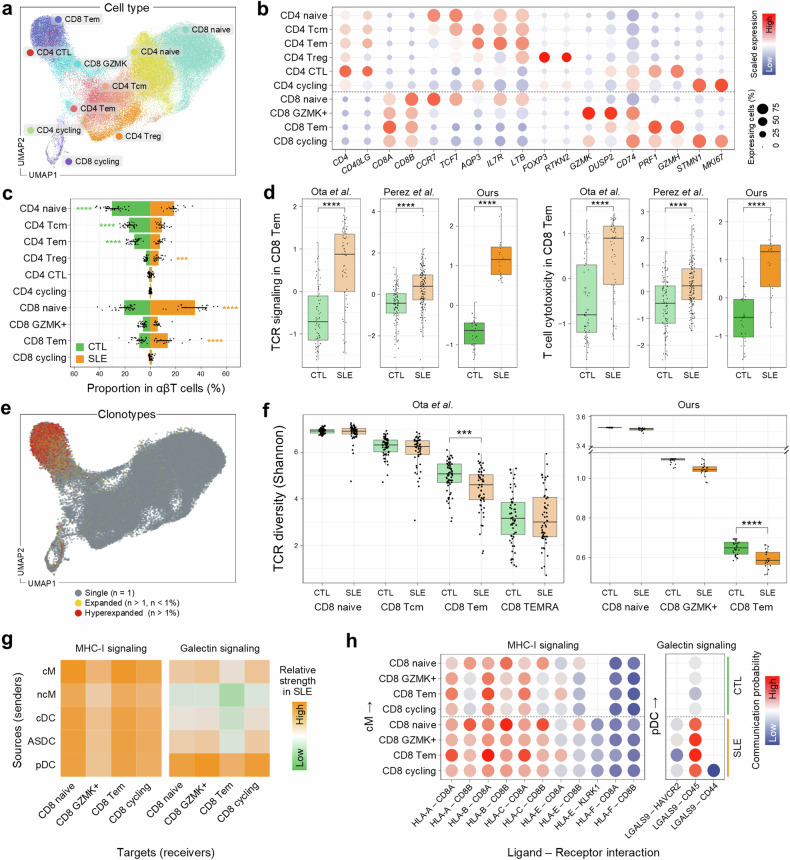
Distinct T cell activation and TCR diversity in SLE. **a** A UMAP visualization for cell composition by αβT cell subtypes: CD4^+^ naive (CD4 naive), central memory (CD4 Tcm), effector memory (CD4 Tem), regulatory (CD4 Treg), cytotoxic (CD4 CTL), cycling (CD4 cycling), CD8^+^ naive (CD8 naive), GZMK^+^ memory (CD8 GZMK), effector memory (CD8 Tem) and cycling (CD8 cycling) T cells. **b** The scaled average expression of marker genes across ten αβT cell subtypes, with dot size indicating the expressing cell proportion and the color gradient showing expression levels. **c** The proportion of αβT cell subtypes by disease state. The lines on the bar indicate the IQR, spanning from Q1 to Q3. Statistically significant increases are marked with orange stars for SLE and green for CTL. **d** Box plots comparing TCR signaling and T cell cytotoxicity in CD8 Tem cells across controls (CTL) and patients with SLE in three studies. The box represents the IQR, spanning from Q1 to Q3, with the line inside the box indicating the median. The whiskers extend to the smallest and largest values within 1.5 times the IQR. **e** A UMAP visualization delineates TCR clonotype distribution across αβT cells. Cells are colored based on clonotype expansion status. **f** Box plots showing TCR diversity (Shannon index) in CD8 T cell subtypes across controls and patients with SLE in two studies: Ota et al.^17^ (left) and our study (right). The box represents the IQR, spanning from Q1 to Q3, with the line inside the box indicating the median. The whiskers extend to the smallest and largest values within 1.5 times the IQR. **g** Heat maps of MHC-I and Galectin signaling strength from myeloid cells to CD8 T cell subtypes. The color gradient represents the relative strength of signaling. **h** Ligand–receptor communication probability of MHC-I (cM to CD8 T) (left) and Galectin signaling (pDC to CD8 T) (right). The color gradient represents communication probability and the empty dots indicate nonsignificant interactions. **P < 0.01, ***P < 0.001 and ****P < 0.0001.

To explore alterations in the phenotype, functional state and clonality of T cells in SLE, we further dissected CD4 and CD8 T cells using established marker genes (Fig. 3a,b). The overall proportion of CD4 T cells decreased in SLE (Fig. 3c). However, the abundance of CD4 regulatory T cells (T_reg_) was notably enriched in SLE, albeit with downregulation of *CTLA4* and *IL2RA* (Supplementary Fig. 8a), implying that their suppressive functionality may be impaired. Consistent with this was the attenuated activity levels of IL-2 and TGF-β signaling pathways in CD4 T_reg_ cells of SLE (Supplementary Fig. 8b), affording a diminished capacity to maintain immune tolerance. Together, these data strongly suggest that the dysfunction of CD4 T_reg_ cells may contribute to the severity of inflammation and autoimmunity in SLE, leading to a less controlled and more aggressive disease course. Moreover, we observed heightened cytotoxic activity of CD4 cytotoxic T cells in SLE (Supplementary Fig. 8c,d), which could exacerbate inflammatory responses and tissue damage, leading to more severe disease manifestations.

To further understand how T cell-mediated cytotoxicity is dysregulated in SLE, we next investigated the CD8 T cell compartment. In contrast to CD4 T cells, the overall proportional abundance of CD8 T cell subsets was increased, particularly in naive and effector memory T (Tem) cells (Fig. 3c). As expected, gene set activity measurements for the canonical CD8 Tem functions (TCR signaling and T cell cytotoxicity) indicated enhanced engagement in immune responses in SLE, confirmed by two independent external datasets (Fig. 3d). In line with this observation, CD8 Tem cells were also specified in clonally expanded clonotypes in SLE (Fig. 3e and Supplementary Fig. 8e) with their lower TCR diversity (Fig. 3f) indicating that the selected T cell clones were repeatedly activated, probably in an autoimmune response with augmented cytotoxic activity. This was consistently observed in an external dataset (Fig. 3f). Moreover, CD4 cytotoxic T cells, which are commensurate with cytotoxic function, showed the selective expansion of CD8 Tem cells with decreased TCR diversity in SLE (Fig. 3e and Supplementary Fig. 8e,f). Together, our data indicated that the clonal expansion of cytotoxic T cells, probably targeting autoantigens, could contribute to the persistent inflammation and chronic tissue damage in SLE, thereby exacerbating disease severity.

To activate CD8 T cells in response to and to communicate with other cells, MHC-I signaling is essential to recognize and eliminate abnormal cells by presenting antigens. Galectin signaling helps regulate their functions, facilitating effective interactions with other immune cells and enhancing coordinated immune responses. To better understand how cellular behaviors of CD8 T cells and their interplay with other cells become altered in SLE, we analyzed cell–cell communication networks across CD8 T cell subtypes using a ligand–receptor interaction knowledgebase^32^. In SLE, CD8 Tem cells exhibited relatively stronger interactions with cM via MHC-I signaling and with pDC via Galectin signaling (Fig. 3g). More specifically, MHC-I signaling was prominent in ligand–receptor interactions of HLA class I molecules with CD8A, CD8B and KLRK1, while Galectin signaling was primarily mediated between LGALS9 (a biomarker in SLE pathogenesis^50^) and CD45 (Fig. 3h). Together, our analysis highlighted the critical impacts of dysregulated CD8 T cells with their aberrant molecular and cellular behaviors in shaping disease outcomes.

### Expansion of clonally restricted cytotoxic CD8 T cell during SLE flare states

**Fig. 4:**
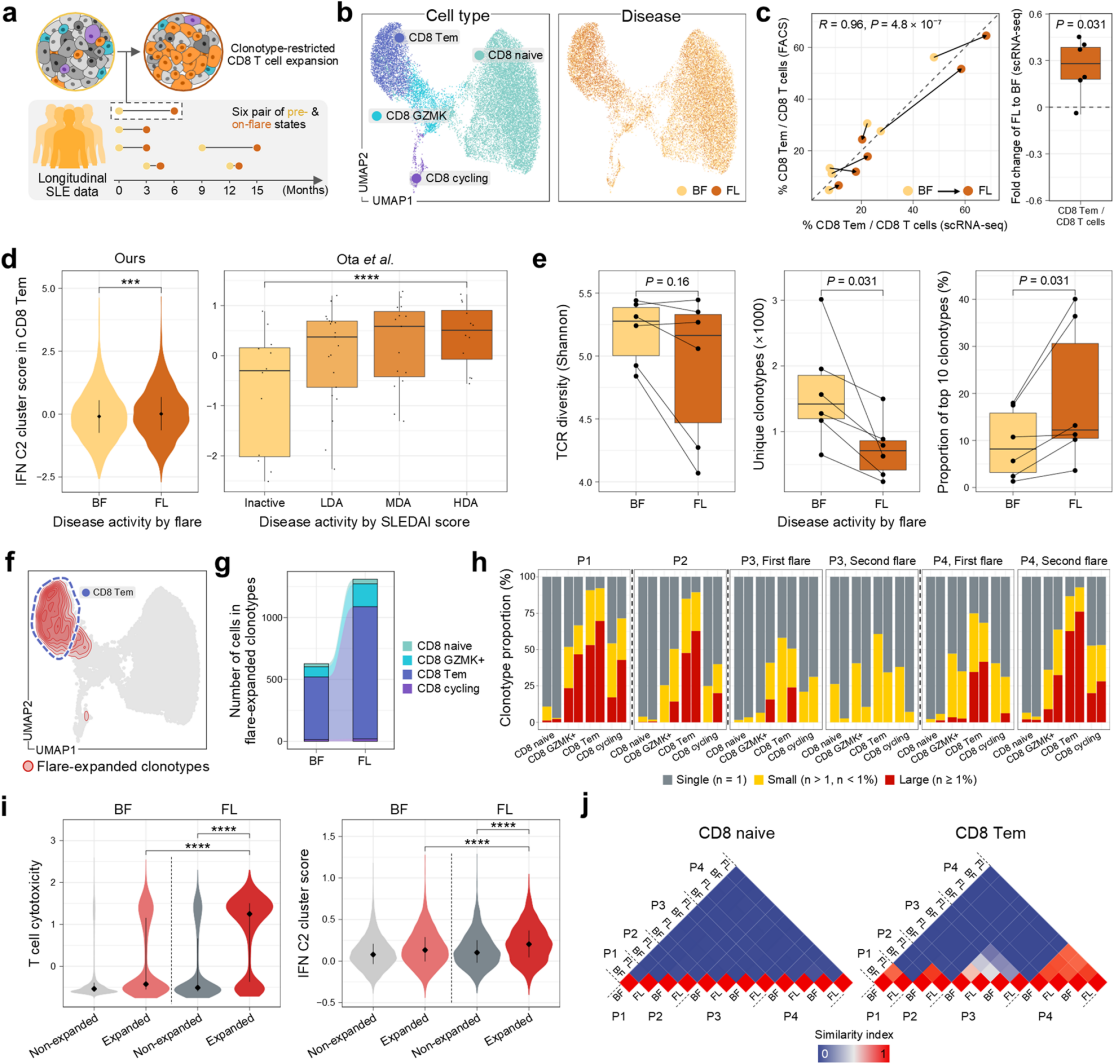
Expansion of clonally restricted cytotoxic CD8 T cell in SLE flare state. **a** A schematic representation of CD8 T cell profiling in SLE flare using scRNA-seq and scTCR-seq. The timeline illustrates longitudinal tracking of BF and FL states in patients. **b** A UMAP visualization for cellular composition of CD8 T cells during flares, categorized by cell types (left) and disease status (right). **c** The correlation between the proportion of CD8 Tem cells among CD8 T cells measured by scRNA-seq and flow cytometry across BF and FL samples (left). A box plot showing the fold change of CD8 Tem cell proportion (FL relative to BF) in scRNA-seq data (right). Each dot represents an individual patient. The box represents the IQR, spanning from Q1 to Q3, with the line inside the box indicating the median. The whiskers extend to the smallest and largest values within 1.5 times the IQR. **d** Plots showing the IFN C2 cluster score in CD8 Tem cells during different states of disease activity. A violin plot for our dataset comparing BF and FL states (left). Lines within the violin indicate the IQR, spanning from Q1 to Q3.A box plot from Ota et al.^17^ across different SLEDAI states (right). The box represents the IQR, spanning from Q1 to Q3, with the line inside the box indicating the median. The whiskers extend to the smallest and largest values within 1.5 times the IQR. HDA, high disease activity; LDA, low disease activity; MDA, moderate disease activity. **e** TCR diversity and clonotype analysis in CD8 T cells by flare status. The plots show TCR diversity (left), the number of unique clonotypes (middle) and the proportion of the top ten clonotypes in CD8 T cells (right), comparing BF and FL states. The lines connecting paired samples from the same patient indicate changes between BF and FL time points. The box represents the IQR, spanning from Q1 to Q3, with the line inside the box indicating the median. The whiskers extend to the smallest and largest values within 1.5 times the IQR. **f** A UMAP visualization showing the density of expanded TCR clonotypes in CD8 T cells during flares. **g** An alluvial plot illustrating the distribution of expanded clonotypes across CD8 T cell subtypes in BF and FL states. **h** Bar plots showing the proportion of TCR clonotype in CD8 T cell subtypes of each pair of BF and FL time points across patients, with clonotypes categorized by color. **i** Violin plots comparing T cell cytotoxicity (left) and IFN C2 cluster scores (right) between expanded and non-expanded clonotypes in BF and FL states. The lines within the violin indicate the IQR, spanning from Q1 to Q3. **j** Heat maps representing the similarity index between samples from different time points in CD8 naive (left) and CD8 Tem cells (right), with patient numbers indicating sample origin. The color gradient represents the similarity index. ***P < 0.001 and ****P < 0.0001.

The hyperactivation of clonally active CD8 T cells in SLE drives inflammation. This exacerbates disease symptoms and results in more severe and unpredictable flares^51^. To capture the functional changes in CD8 T cells over the course of disease progression, we collected and analyzed before-flare (BF) and on-flare (FL) states (total, *n* = 12) (Fig. 4a and Supplementary Table 1). The flare state showed a significant increase in the SLEDAI score compared with the baseline pre-flare state, along with a concomitant rise in proteinuria (Supplementary Fig. 1b) and elevated expression of cytotoxic genes (Supplementary Fig. 9a), underscoring the systemic inflammatory burden during flares^52^.

We identified CD8 T cell subtypes across BF and FL samples (Fig. 4b). As expected, CD8 Tem cells were strongly associated with the flare state. This increase was also confirmed by flow cytometry, indicating a consistent expansion during flares (Fig. 4c and Supplementary Fig. 10). This observation aligns with the analysis of CD8 T cell differentiation trajectory, showing that the more differentiated cells, CD8 Tem, were more prevalent during flares and exhibited a higher level of cytotoxicity (Supplementary Fig. 9b,c). To further characterize CD8 Tem cells in terms of their IFN-related response during flare states, we estimated the gene set activity of IFN C2 cluster genes, which were relevant to lymphocytes and more responsive to shifts in disease activity (Fig. 1e). Intensified IFN activity was found in flares relative to pre-flare states (Fig. 4d). Moreover, we could derive a consistent trend of increased IFN activity in patients with higher SLEDAI scores from an independent dataset (Fig. 4d)^17^, indicating that heightened IFN activity in CD8 Tem cells contributes to exacerbating inflammation and worsening disease activity during flares.

To understand further how the immune system adapts and evolves during flares, we sought to identify which CD8 T cell clones actively expand as the disease progresses. To achieve this, we analyzed TCR sequences in CD8 T cells and tracked their clonotypes throughout the flare states. The overall reduction in TCR diversity, coupled with a decrease in unique clonotypes and an expanded proportion of the top ten enriched clonotypes, was observed during flares (Fig. 4e and Supplementary Fig. 11a). This shift indicates a diminished range of antigens that CD8 T cells recognize and respond to, highlighting more targeted but potentially more aggressive autoimmune activity during flares.

We next tracked specific clones that expanded during flare states. Certain clonotypes exhibited more than a twofold expansion during flares compared with pre-flare states (Fig. 4f and Supplementary Fig. 11b). These clonotypes, which were predominantly naive cells before flare onset, transitioned to effector phenotypes during flares (Fig. 4g). Additionally, the proportion of large clonotypes significantly increased during flares, reflecting greater clonal dominance within the CD8 T cell repertoire (Fig. 4h). Notably, these flare-expanded clonotypes demonstrated elevated expression of cytotoxicity-related genes and heightened activity of IFN C2 cluster genes relative to other clonotypes, with both metrics significantly increased from pre-flare levels (Fig. 4i). These findings suggest that flare-expanded clonotypes play a central role in mediating immune responses during flares and are strongly associated with disease exacerbation. Furthermore, we evaluated the similarity of CD8 TCR clonotypes across patients and flare states to investigate potential correlations with autoantibody responses. While TCR clonotypes varied between patients, individual patients showed consistent clonotype profiles across flare states, with greater similarity observed in CD8 Tem compared with CD8 naive T cells (Fig. 4j).

## Discussion

In this study, we utilized high-resolution scRNA-seq of serial PBMCs from patients with SLE to provide novel insights into the immune dysregulation and dynamic interactions between immune cells during longitudinal monitoring. Our findings reaffirmed previously reported alterations in cellular composition in SLE, including a decrease in CD4 T cells and an increase in CD8 T cells and B cells^10,11^. We further confirmed the pronounced type I IFN signatures in multiple cell clusters. Additionally, by analyzing IFN modules in a cell type-specific manner, we stratified patients with SLE into four distinct molecular subtypes, not only in our scRNA-seq data but also in two independent external datasets^11,17^. Notably, M5.12 module^46^, which correlates strongly with renal flares and overall disease activity in ref. ^46^, was predominantly overlapped with our cluster C2, which is enriched in CD8 T lymphocytes. This suggests that C2 cluster genes in CD8 T cells appear to be more sensitive to changes in disease activity and may serve as a more precise marker for tracking disease progression and flare-ups, a hypothesis that requires further validation in future studies.

In particular, we revealed a significant increase of ABCs with the most pronounced IFN C1 cluster module scores among B cell subtypes. The ratio of ABCs to memory B cells with differential TF activity was also prominently elevated in our data as well as in previous scRNA-seq data^11^, underscoring their potential roles in SLE pathogenesis by promoting cell proliferation and differentiation into plasma cells. This might also imply a reduced BCR signaling threshold, potentially elevating autoantibody production^53^. Moreover, we observed increased gene expression and TF activity of STAT1 in ABCs. This may reflect an upregulated IFN response, as previously reported in patients with SLE^54,55^. However, STAT1 is not merely a downstream mediator; it also plays an active role in propagating immune responses through the induction of IFN-stimulated genes and formation of feed-forward loops^56^. In support of this, functional studies have shown that STAT1 phosphorylation at a specific site is essential for the development of lupus-like autoimmunity in murine models^57^, supporting its potential pathogenic role. Therefore, the increased STAT1 activity we observed may be both a consequence of IFN signaling and a driver of sustained autoimmune activation. Clarifying this relationship will require further experimental studies using lupus-prone animal models with B cell-specific manipulation of STAT1. Furthermore, given that STAT1 is activated downstream of JAK kinases, our observation of enhanced STAT1 activity in ABCs supports the rationale for JAK inhibition as a potential therapeutic strategy in SLE. By suppressing this pathway, JAK inhibitors may help reduce ABC-driven immune activation and inflammation during disease flares.

T cells are pivotal drivers in SLE pathogenesis. They modulate B cell responses, influencing autoantibody production and initiating inflammation. Consequently, their infiltration into target tissues leads to chronic tissue damage^58^. Dysregulated T cell signaling exacerbates these processes, resulting in more severe disease manifestations. Our findings highlighted the augmented cytotoxicity and clonal proliferation of CD8 Tem cells in SLE, marked by intensified cell-to-cell interactions and elevated expression of ISGs. This suggests the critical involvement of specific T cell subsets in SLE progression with type I IFN signaling stimulation, and thereby presents a compelling rationale for therapeutic interventions targeting such cell populations.

We also examined the cellular and molecular changes during acute SLE exacerbations, particularly for CD8 T cells. This novel approach uncovered a significant expansion of CD8 Tem cells, characterized by upregulated expression of cytotoxicity-associated genes and intensified IFN activity during flare-ups, indicating that CD8 Tem cells contribute to exacerbating inflammation and worsening disease activity. Notably, TCR repertoire analysis further identified diverse and unique clonotypes among these cells in each flare event. We observed an overall reduction in TCR diversity and an expanded proportion of the top ten enriched clonotypes during flares. Additionally, the clonal expansion during flares was prominent in CD8 Tem cells. Prior studies have shown that clonally expanded CD8 Tem cells in lupus nephritis directly mediate tissue injury through epithelial adhesion and tubulitis^59^, suggesting that expanded CD8 Tem cells may play a direct role in driving renal inflammation and damage. These findings suggest the observed clonal expansion and cytotoxic signature of CD8 Tem cells in our study may reflect functional involvement in tissue damage.

Together, our longitudinal tracking of functional and clonal dynamics further emphasized the pivotal role of CD8 Tem cells in fueling autoimmune activity throughout disease progression. The increased cytotoxicity of CD8 T cells might expedite this process, potentially resulting in swift inflammatory reactions and the convergence of antigens during flare-ups. Despite these findings, we did not identify specific pathogenic clonotypes within the expanded CD8 Tem cell population, probably due to limited patient numbers and sampling time points. Furthermore, diversity measurements may have been sensitive to the thresholds used in defining clonotype expansions, potentially amplifying the significance of rare clonotypes. Nevertheless, CD8 T cell clonal tracking provides cell-specific and temporally dynamic resolution that may more effectively capture imminent immune activation and inform real-time therapeutic decisions, whereas conventional biomarkers often lack the precision to identify disease-driving immune subsets. Therefore, future studies with larger cohorts and deeper longitudinal sampling are needed to validate these findings and to explore the potential of clonal dynamics as predictive markers and therapeutic targets in SLE.

While our study advances the understanding of SLE pathogenesis, it is limited in direct observations of immune responses within tissues and in the sample size. The juxtaposition of single-cell profiles from PBMCs and kidney biopsies could provide a comprehensive view of immune cell dysregulation and their cellular engagement with the kidney parenchyma and stroma, particularly in lupus nephritis. Furthermore, this dual analysis could facilitate understanding the roles of circulating versus tissue-resident immune cells in lupus nephritis, providing insights into targeted interventions^60^. Despite such expected advantages, kidney biopsies are invasive and difficult to obtain, necessitating careful patient selection and clinical justification. With a relatively straightforward and minimally invasive procedure, our study serially collected PBMCs from patients with SLE throughout the disease course and enabled us to identify surrogate bloodstream biomarkers with great potential for clinical utility. In regard to the constrained sample size in this study, we corroborated our observations in two independent external datasets^11,17^, and future research with larger cohorts would reinforce our findings and translate them to designing therapeutic interventions.

In summary, our study elucidates the intricate cellular dynamics and immune alterations in SLE, which may serve as potential biomarkers for predicting disease trajectories in patients. Molecular characterization of CD8 Tem cells and their TCR clonotypes during flare events establishes a critical step toward expanding our understanding of immune repertoire monitoring, and may also provide a foundation for the development of the next generation of therapeutic interventions in the management of SLE.

## Supplementary information

Supplementary Information

Supplementary Table 1

Supplementary Table 2

Supplementary Table 3

Supplementary Table 4

Supplementary Table 5

## Data Availability

The raw and processed single-cell sequencing data generated in this study are available from the Gene Expression Omnibus (GEO) under accession number GSE254176. To support comparative analyses, external datasets were accessed via the NBDC Human Database (E-GEAD-397) and the GEO database (GSE174188). All codes supporting the findings of this study are available from the corresponding authors upon request.
