# Supplementary material for: Tracking clonal dynamics of CD8 T cells and immune dysregulation in progression of systemic lupus erythematosus with nephritis
Source: Exp Mol Med. 2025 Aug 1;57(8):1700–10. doi: 10.1038/s12276-025-01504-2 (PMC12411632; doi:10.1038/s12276-025-01504-2)
Supplement: Supplementary file 1 — Supplementary Information [file 12276_2025_1504_MOESM1_ESM.pdf]

Supplementary information

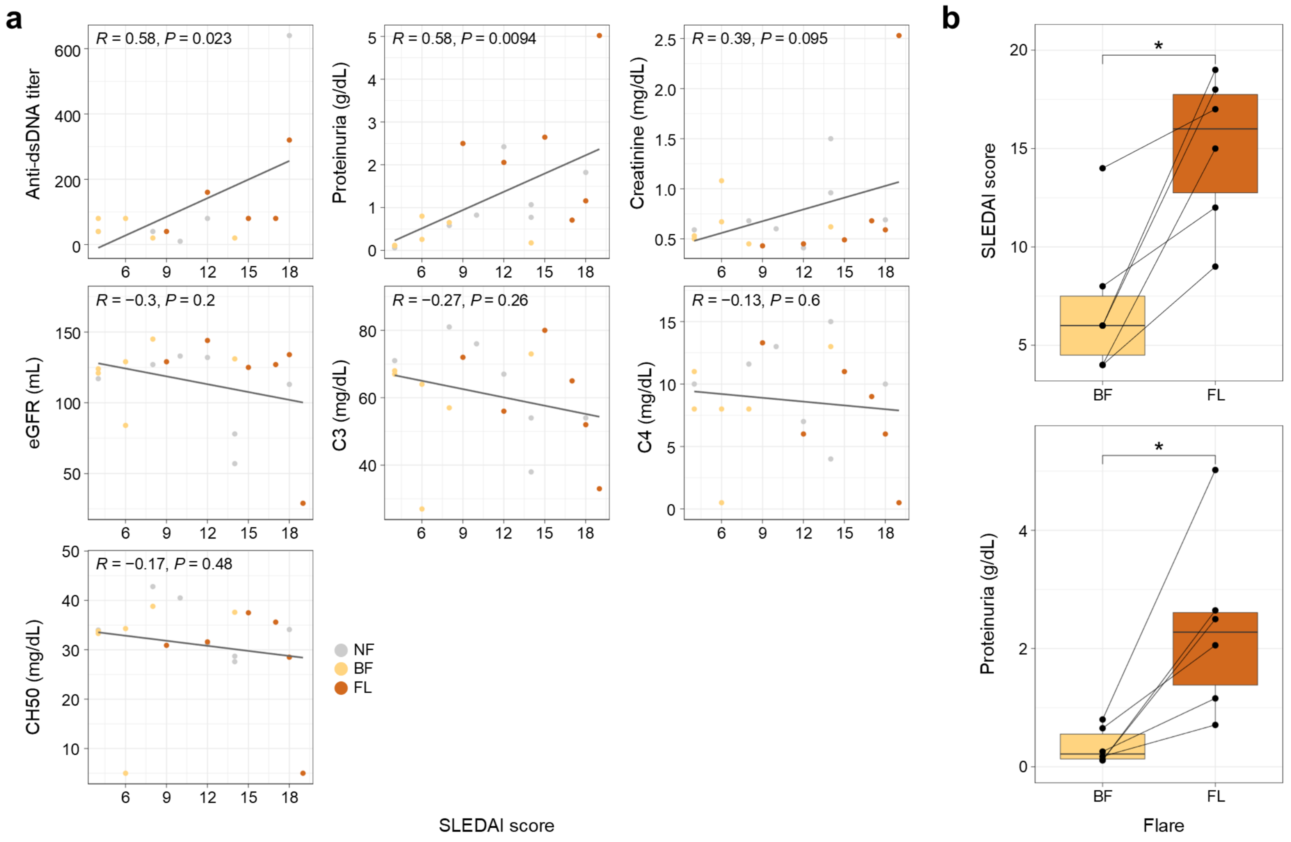

Supplementary Fig. 1: Association of clinical markers with disease activity in SLE.

a Scatter plots showing the correlation between the Systemic Lupus Erythematosus Disease Activity Index (SLEDAI) score and various clinical markers in patients with SLE. Clinical markers include anti-dsDNA titer, proteinuria, creatinine, estimated glomerular filtration rate (eGFR), complement components C3 and C4, and CH50 (50% Hemolytic Complement). The lines represent linear regression fits for each marker against the SLEDAI score. Colors represent different disease states. NF, Non-flare state; BF, pre-flare state; FL, on-flare state. **b** Box plots comparing SLEDAI score levels (top) and proteinuria (bottom) between BF and FL states. Lines connecting paired samples from the same patient indicate changes between BF and FL timepoints. *, $P < 0.05$.

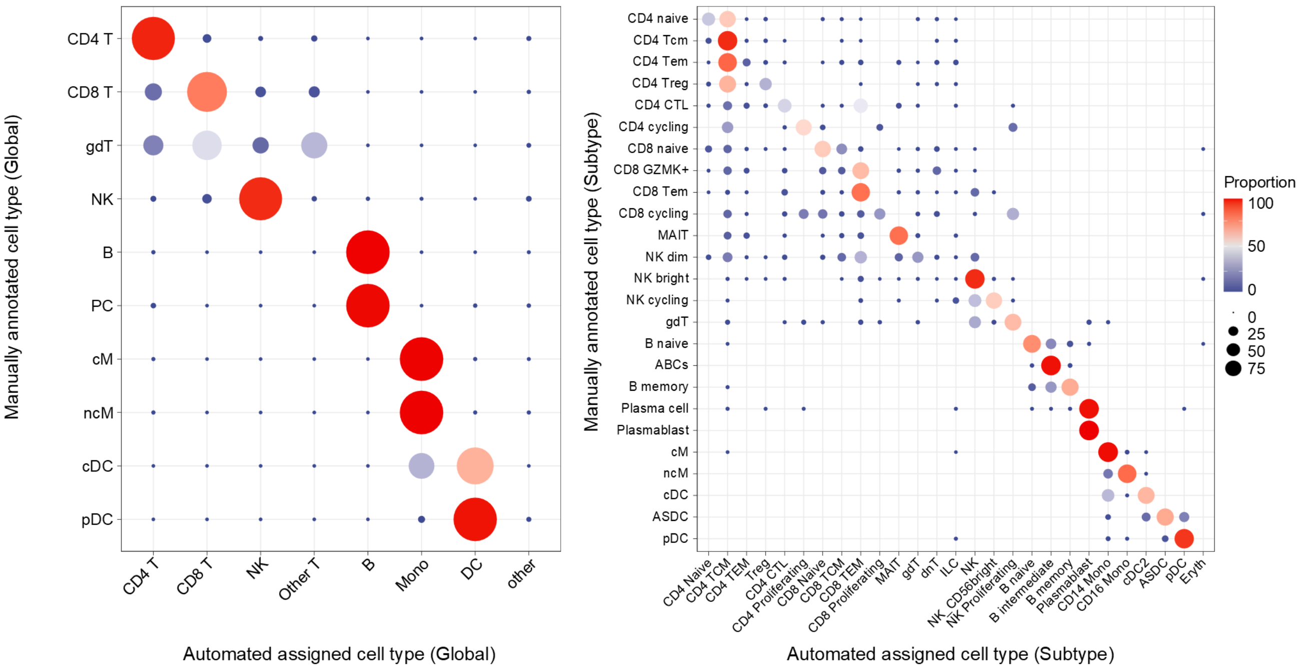

Supplementary Fig. 2: Correlation between manual and automated cell type annotation.

Dot plots showing the agreement rates between manual annotation of cell types and automated assignment by Azimuth at a global (left) and subtype level (right). Cell types with the matched label between manual annotation and automated assignment are highlighted with agreement rate values. Both dots size and the colors represent the magnitude of agreement rates.

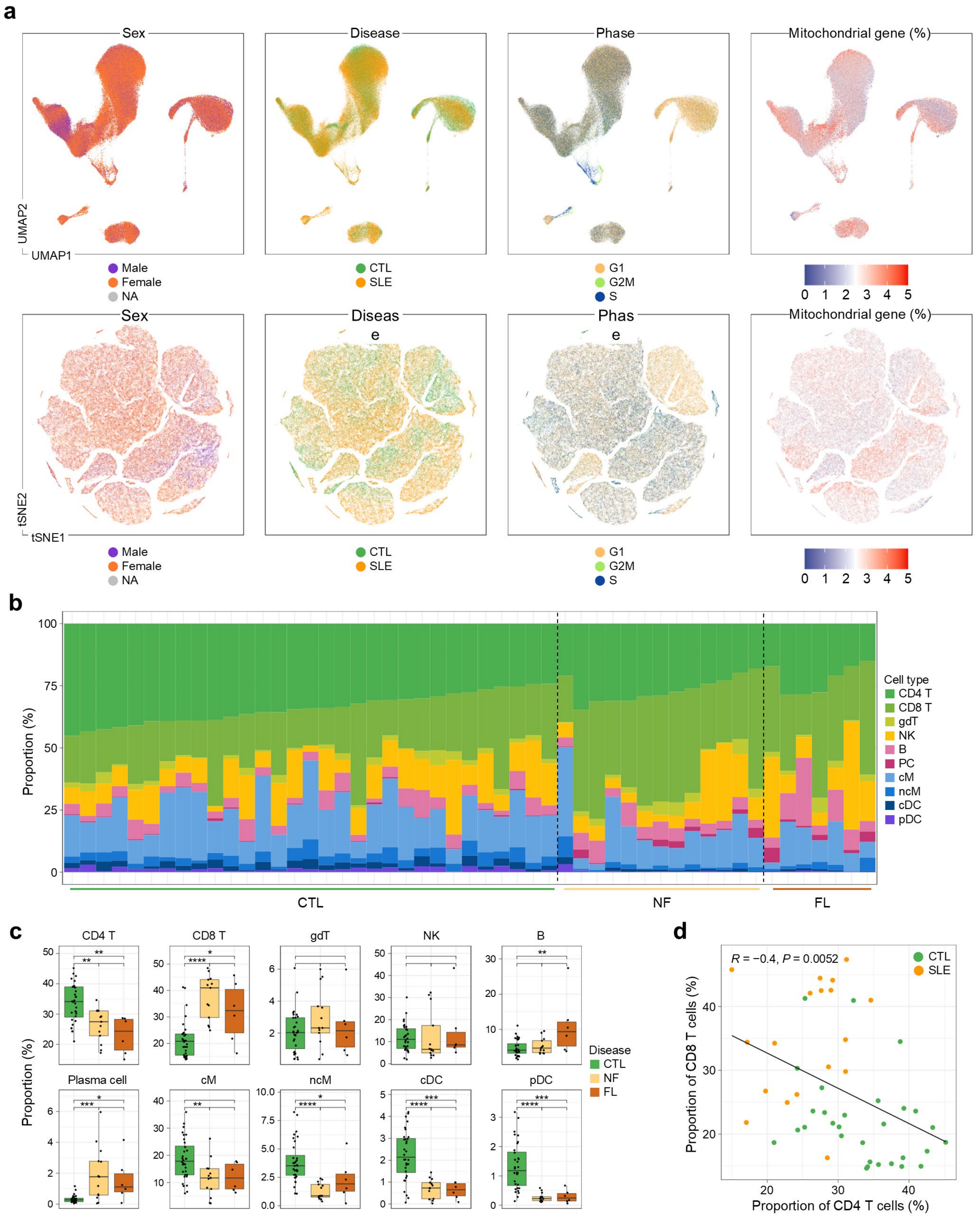
a
Sex
Disease
Phase
Mitochondrial gene (%)
UMAP2
UMAP1
Male
Female
NA
CTL
SLE
G1
G2M
S
0 1 2 3 4 5
tSNE2
tSNE1
b
Proportion (%)
Cell type
CD4 T
CD8 T
gdT
NK
B
PC
cM
ncM
cDC
pDC
CTL
NF
FL
c
CD4 T
CD8 T
gdT
NK
B
Plasma cell
cM
ncM
cDC
pDC
Proportion (%)
Disease
CTL
NF
FL
d
R = −0.4, P = 0.0052
CTL
SLE
Proportion of CD8 T cells (%)
Proportion of CD4 T cells (%)

Supplementary Fig. 3: Comprehensive analysis of immune cell composition in SLE.

a UMAP (top) and t-SNE (bottom) visualizations showing the heterogeneity of cell composition within PBMCs, influenced by sex, disease status, cell cycle phase, and mitochondrial gene expression percentage. Colors in the UMAP and t-SNE plots correspond to categories within each factor. **b** Bar plot representing the proportion of total immune cell types across individual samples, categorized by disease state. **c** Box plots illustrating the distribution of each immune cell type proportion across different disease states. **d** Scatter plot showing the relationship between CD4 and CD8 T cell proportions in CTL and SLE. Colors of dots represent disease states and linear regression lines are fitted for each T cell proportion. *, $P < 0.05$; **, $P < 0.01$; ***, $P < 0.001$; ****, $P < 0.0001$.

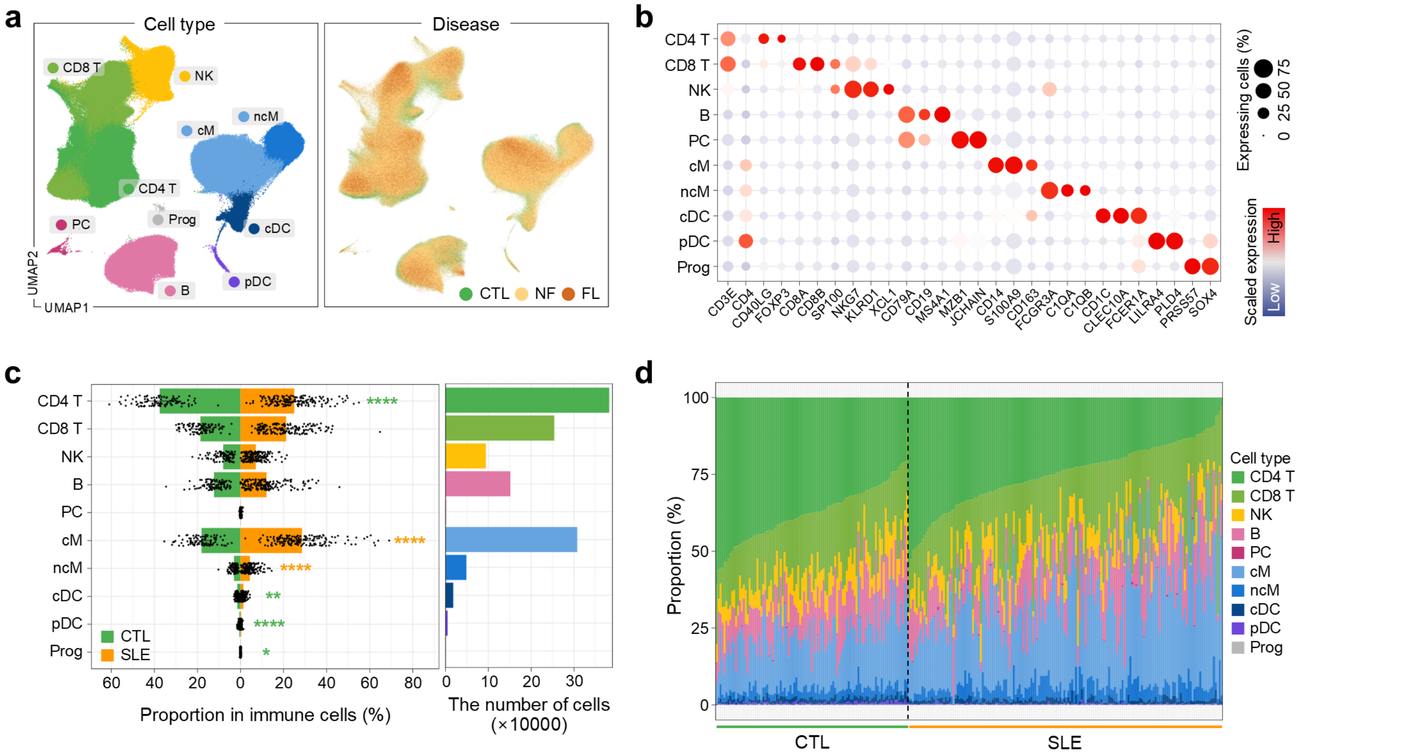

Supplementary Fig. 4: Characterization of immune cell landscape in external SLE scRNA-seq data.

a UMAP visualization for cell composition by cell types (left) and disease activity (right) of total cells in external SLE scRNA-seq data. Prog, progenitor cells; **b** Scaled average expression of marker genes across 10 cell types in total immune cells of the external SLE dataset. The color gradient represents expression levels, and the size of each dot indicates the proportion of expressing cells in each cell type. **c** Bar plot illustrating the proportion of immune cell types in PBMCs by disease state (left) and the total number of immune cells (right) in the external SLE dataset. Orange stars signify statistically significant increases in SLE, while green indicates increases in CTL. **d** Bar plot representing the proportion of total immune cell types across individual samples in the external SLE dataset. *, $P < 0.05$; **, $P < 0.01$; ****, $P < 0.0001$.

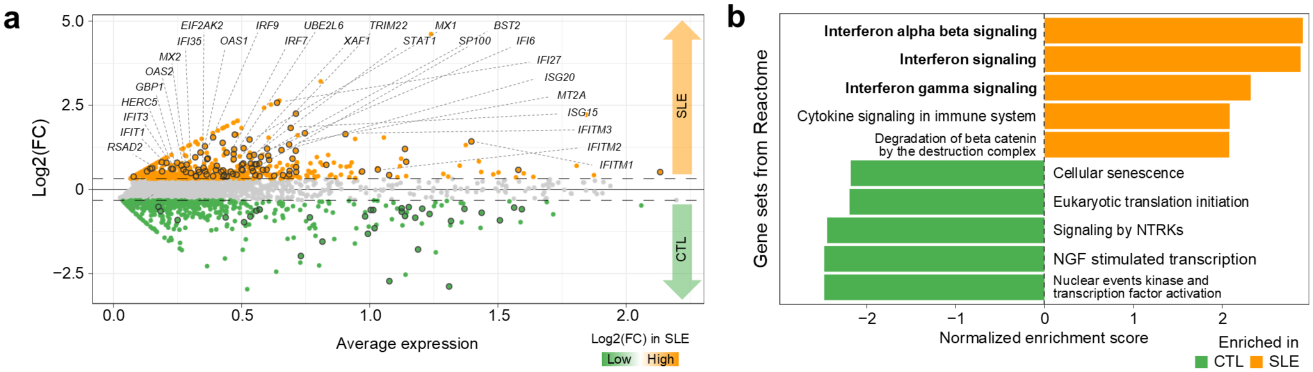

Supplementary Fig. 5: Differential expression and pathway analysis in SLE.

a MA plot illustrating differentially expressed genes in total SLE cells compared to controls. The y-axis represents the log2 fold change (FC) in expression, while the x-axis shows the average expression of genes. Gray dashed lines represent a fold change cutoff (>1.25). Dots represent individual genes, with colors indicating the significant genes in each group. Circles on the plot indicate genes associated with enriched gene sets. **b** Bar plot displaying the top 10 gene sets from Reactome, ranked by the lowest false discovery rate. Bars are colored by the direction of enrichment and pathways related to IFN signaling are highlighted in bold.

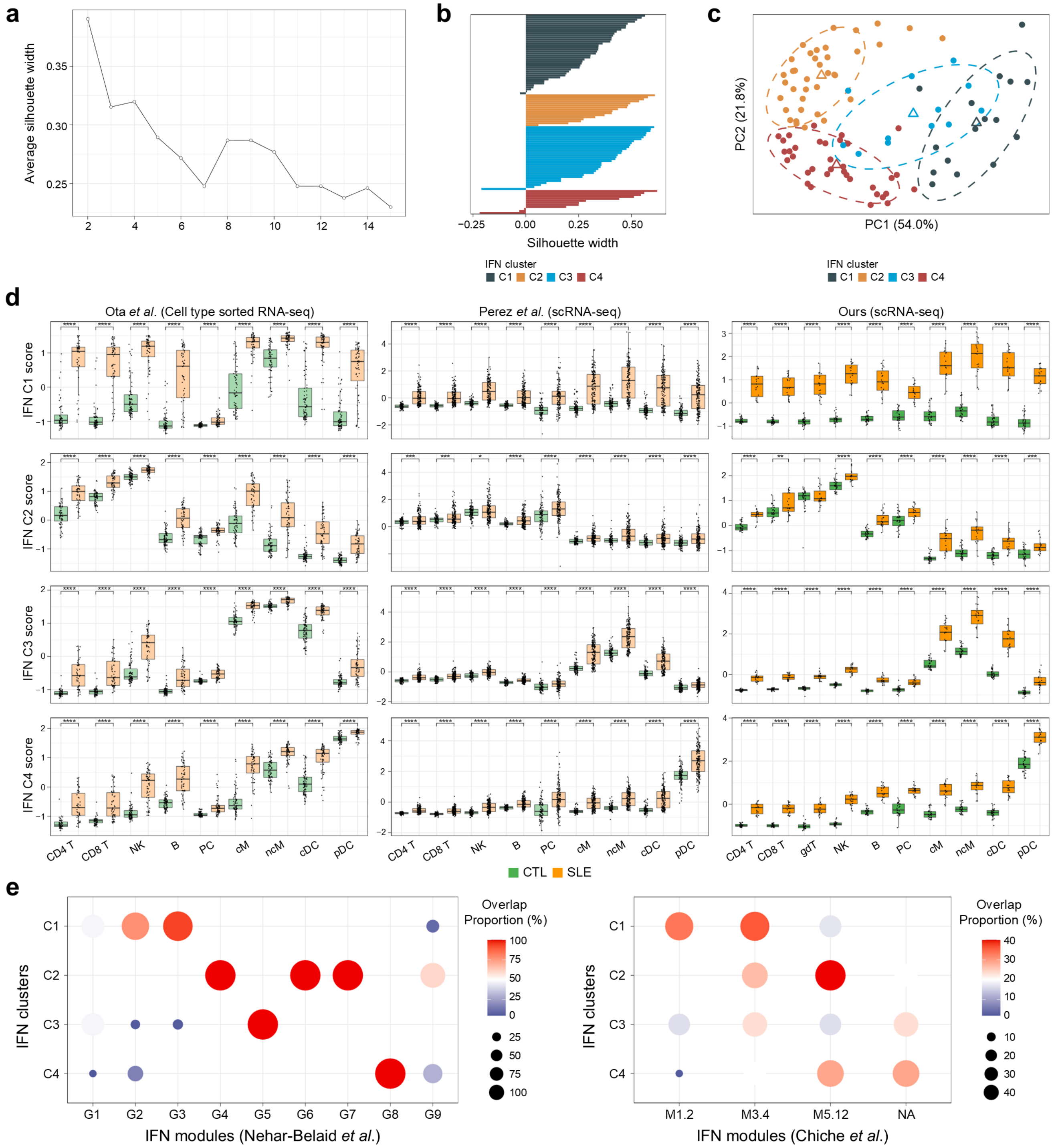

Supplementary Fig. 6: Comparison of IFN clustering with existing IFN signatures.

a Average silhouette width for different numbers of IFN clusters. Higher values indicate better-defined clusters. **b** Silhouette width for individual IFN clusters optimized for four clusters. **c** PCA plot showing the clustering of IFN modules. Colors represent different IFN module

classifications. Dashed lines indicate the boundaries of each cluster at ellipse level 0.8. Triangles represent the mean of each cluster. **d** Box plots showing IFN cluster scores across various immune cell types in CTL and SLE. The data is presented from three studies: Ota *et al*. (left), Perez *et al*. (middle), Our study (right). **e** Bubble plots showing the overlap proportions between IFN clusters identified in this study (C1-C4) and IFN modules identified by Nehar-Belaid *et al*. (left) and Chiche *et al*. (right). The size of the bubbles and the color represents the proportion of overlap.

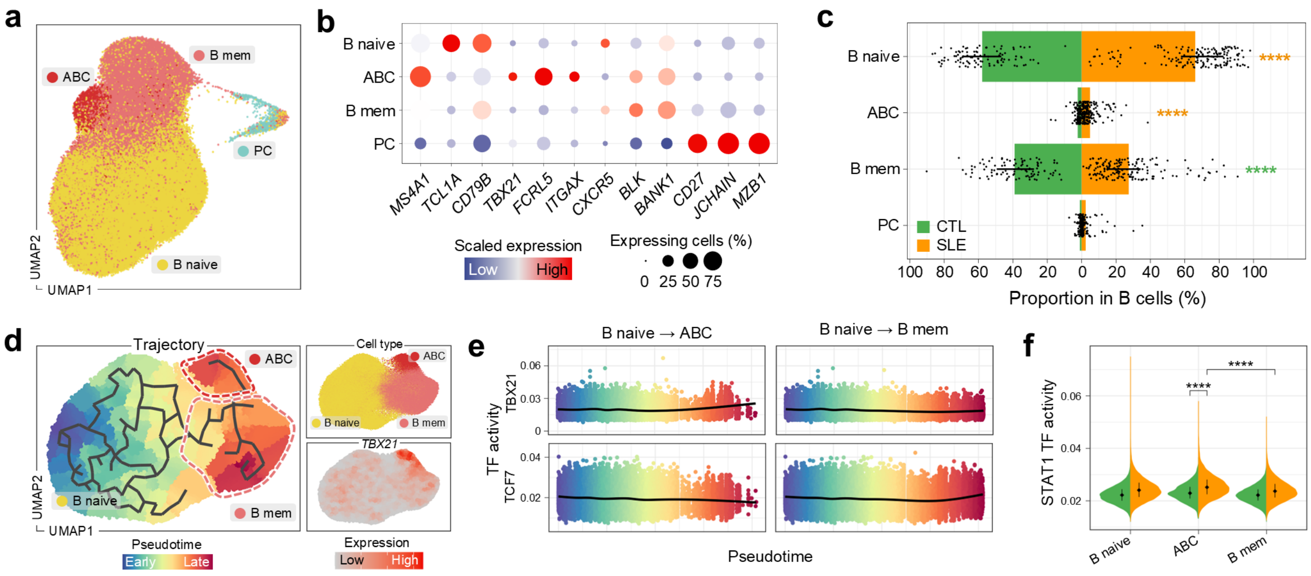

Supplementary Fig. 7: B Cell subtype dynamics in external SLE scRNA-seq data.

a UMAP visualization showing the composition of B cell subtypes (left) and their distribution across disease states (right) in the external SLE scRNA-seq data. **b** Scaled average expression of marker genes across four B cell subtypes. The color gradient represents expression levels, and the size of each dot indicates the proportion of expressing cells in each cell type. **c** Bar plot showing the proportion of B cell subtypes by disease state in the external SLE dataset. **d** (left) UMAP plot illustrating the inferred differentiation trajectory of non-plasma B cells, color-coding from early (blue) to late (red) stages. (right) UMAP plots for subtypes and *TBX21* expression levels. The color gradient indicates expression level. **e** TF activity changes across pseudotime for B cell subtypes in B naive to ABC (left) and B naive to B mem (right). The color gradient represents the progression through pseudotime, with the lines indicating the trend of TF activity. **f** Violin plots of STAT1 TF activity in non-plasma B cell subtypes. Lines within the violin indicate the IQR, spanning from Q1 to Q3. ****, $P < 0.0001$.

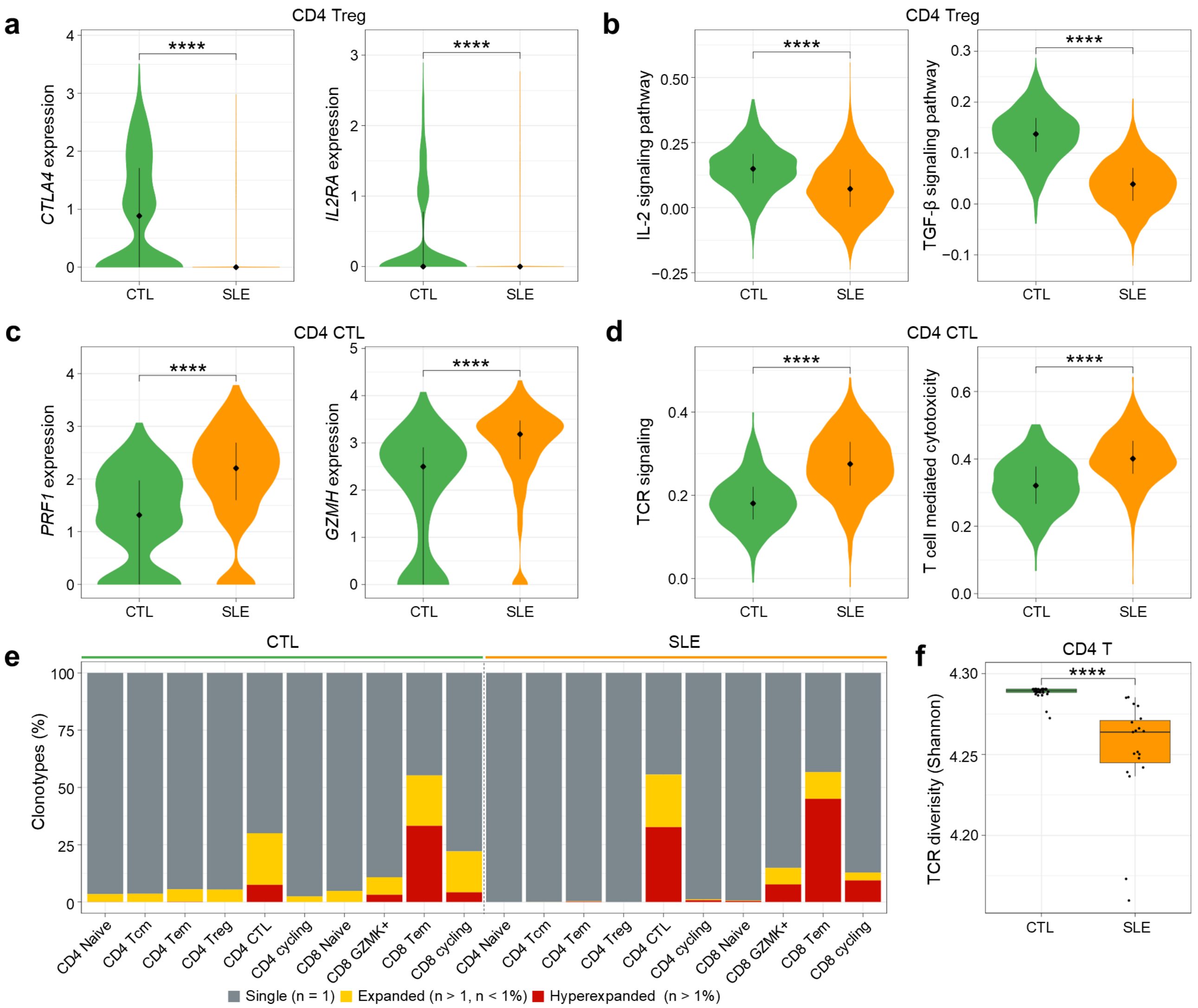

Supplementary Fig. 8: Comparative functional analysis and TCR clonotype profiling in SLE T cells.

a Violin plots showing *CTLA4* and *IL2RA* expression levels in CD4 Treg cells from CTL and SLE. **b** Violin plots representing signaling levels for IL-2 and TGF-β pathways within CD4 Treg cells from CTL and SLE. **c** Violin plots showing *PRF1* and *GZMH* expression levels in CD4 CTL cells from CTL and SLE. **d** Violin plots representing signaling levels for T cell-mediated cytotoxicity and TCR signaling within CD4 CTL cells from CTL and SLE. For panels from **a** to **d**, the line within each violin indicate the IQR, spanning from Q1 to Q3, and the central dot represents the median. **e** Bar plot illustrating the proportion of clonotype expansion within T cell subtypes, contrasting CTL and SLE. Colors indicate different levels of clonotype expansion. **f**

TCR diversity of CD4 T cells using the Shannon diversity index, showing a comparison between control and SLE. ****, $P < 0.0001$.

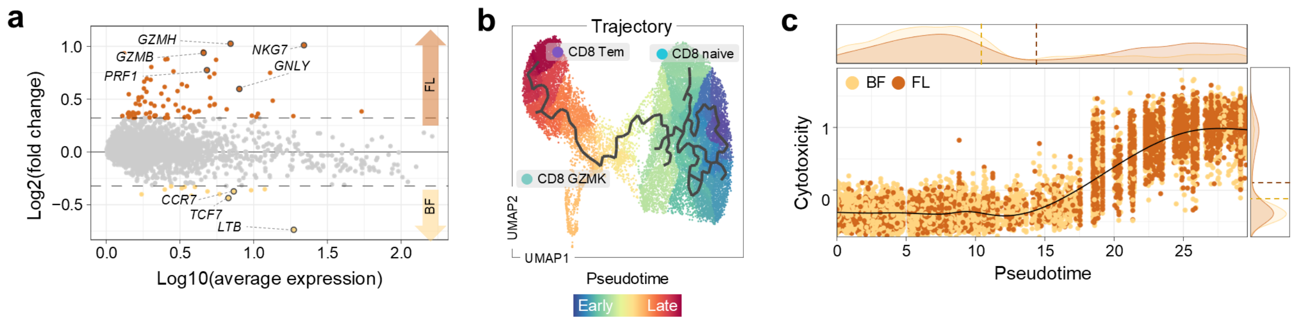

Supplementary Fig. 9: Cytotoxic signature of CD8 T cells during SLE flare state.

a MA plot showing differently expressed genes in CD8 T cells during FL compared with BF. The y-axis represents the log2 fold change in expression, while the x-axis shows the average expression of genes. Gray dashed lines represent a fold change cutoff (>1.25). Dots represent individual genes, with colors indicating the significant genes in each group. **b** UMAP plot with an inferred trajectory showing the differentiation of CD8 T cells from naive to Tem subtypes. The trajectory line and color gradient indicate the progression from early to late differentiation stages. **c** Scatter plot illustrating the correlation between inferred pseudotime and cytotoxicity in CD8 T cells. Data points represent individual CD8 T cells from BF and FL states. A smoothed line shows the trend of cytotoxicity across pseudotime. Ridge plots at the top and right display the density distributions of CD8 T cells for pseudotime and cytotoxicity with dashed lines indicating the mean values for each group.

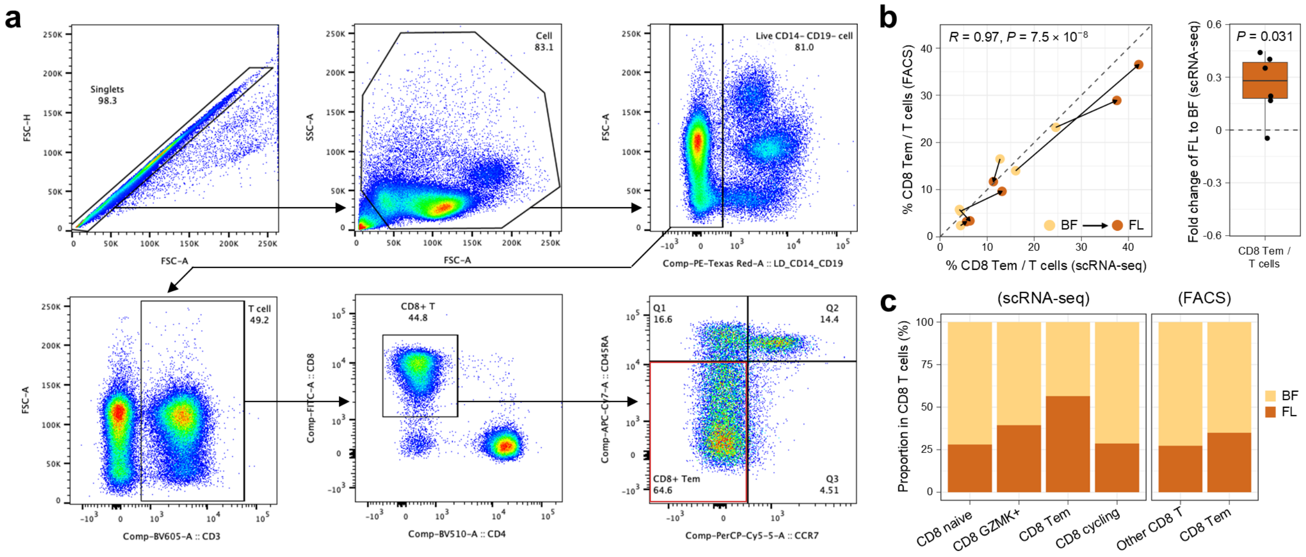

Supplementary Fig. 10: Quantification of CD8 Tem cells using flow cytometry and scRNA-seq across BF and FL states.

a Representative flow cytometry gating strategy from patient P1 timepoint 1 (BF) for identifying $CD8^+$ Tem cells. Sequential gating was performed to select single, viable, $CD14^-CD19^-CD3^+CD8^+$ T cells, followed by $CD4^-CD8^+CCR7^-CD45RA^-$ Tem cell identification. **b** (left) Correlation between the proportion of CD8 Tem cells among T cells measured by scRNA-seq and flow cytometry across BF and FL samples. (right) Box plot showing the fold change of CD8 Tem cell proportion (FL relative to BF) in scRNA-seq data. Each dot represents an individual patient. The box represents the IQR, spanning from Q1 to Q3, with the line inside the box indicating the median. The whiskers extend to the smallest and largest values within 1.5 times the IQR. **c** Distribution of CD8 T cell subtypes across BF and FL states, measured by both scRNA-seq (left) and flow cytometry (right).

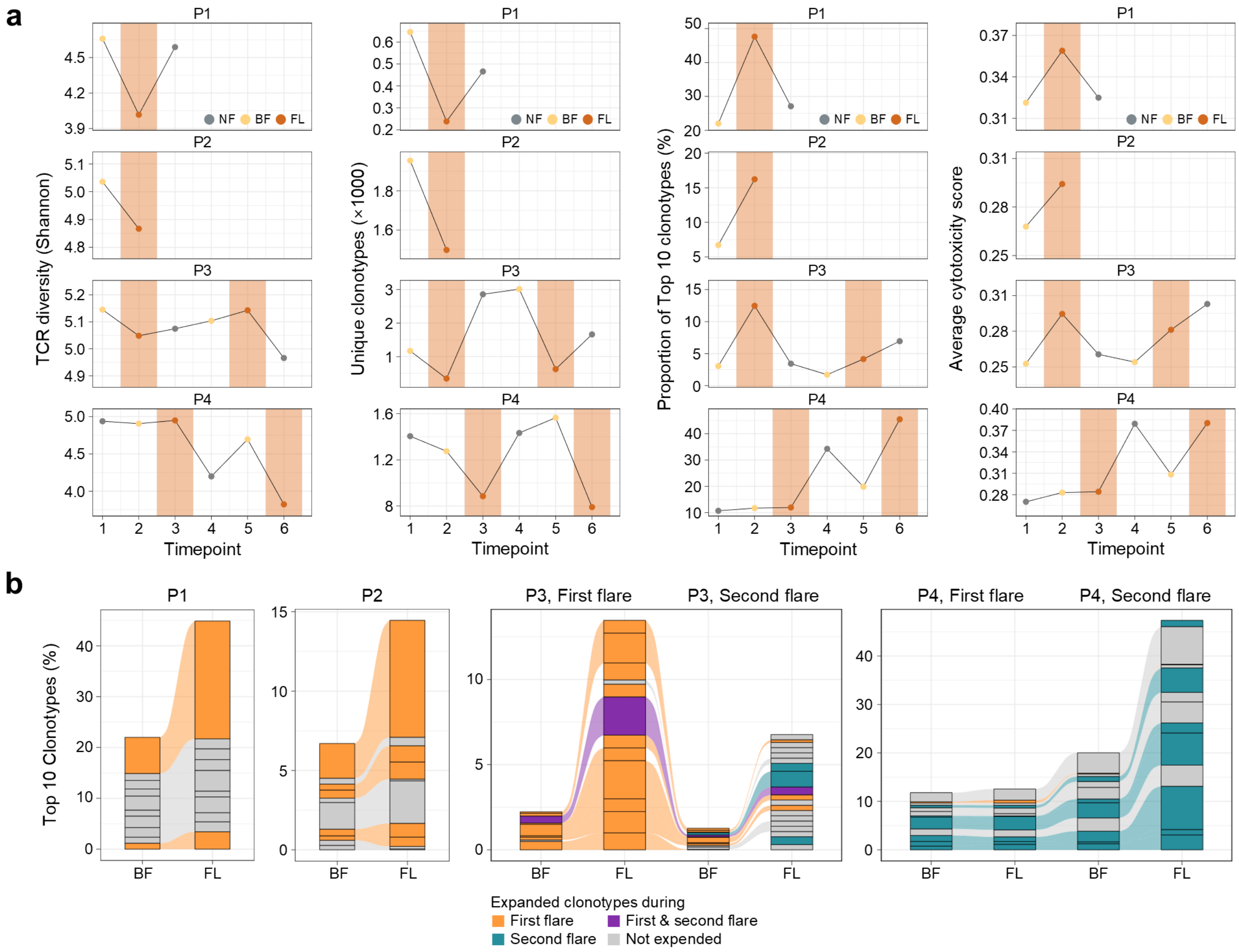

Supplementary Fig. 11: Dynamics of TCR clonotype expansion along with SLE timepoint.

a Temporal changes in TCR clonotypes across SLE timepoints depicted for patients P1 through P4. Each panel illustrates, from left to right, TCR diversity (Shannon index), the count of unique TCR clonotypes, proportion of the top 10 dominant clonotypes, and average levels of T cell-mediated cytotoxicity. These metrics are tracked across SLE progression, with timepoints colored to indicate the presence of flare state. **b** Alluvial plot showing the percentage of top 10 clonotype in CD8 T cells across different timepoints. Each color represents the expansion of specific clonotypes during flare states, with the width of the flow indicating the proportion of each clonotype.
